# Delayed primary fascia closure of Björck grade 4 open abdomen with enteroatmospheric fistulas after repeated surgery for adhesive small bowel obstruction: a case report

**DOI:** 10.1186/s12893-021-01329-6

**Published:** 2021-08-26

**Authors:** Yoshimasa Akashi, Koichi Ogawa, Kaoru Sasaki, Jaejeong Kim, Tsuyoshi Enomoto, Katsuji Hisakura, Yusuke Ohara, Yohei Owada, Kazuhiro Takahashi, Osamu Shimomura, Shinji Hashimoto, Mitsuru Sekido, Tatsuya Oda

**Affiliations:** 1 Department of Gastrointestinal and Hepato-Biliary-Pancreatic Surgery, Faculty of Medicine, University of Tsukuba, 1-1-1 Tennodai, 305-8575 Tsukuba, Ibaraki Japan; 2 Department of Plastic Surgery, Faculty of Medicine, University of Tsukuba, 1-1-1 Tennodai, 305-8575 Tsukuba, Ibaraki Japan

**Keywords:** Open abdomen, Enteroatmospheric fistula, Björck classification, Small bowel obstruction, Case report

## Abstract

**Background:**

An open abdomen with frozen adherent bowels is classified as grade 4 in Björck’s open abdomen classification, and skin grafting after wound granulation is a typical closure option. We achieved delayed primary fascia closure for a patient who developed open abdomen with enteroatmospheric fistulas due to severe adherent small bowel obstruction. We present here the details of his management.

**Case presentation:**

A 52-year-old man suffered acute abdominal pain during a flight and received an emergency laparotomy due to adhesive small bowel obstruction. Repeated laparotomies were required, and later open abdomen and proximal site jejunostomy were selected. After negative pressure wound therapy, he was transferred to our institution. Two enteroatmospheric fistulas emerged on the exposed intestine, and we diagnosed the condition as a Björck grade 4 open abdomen. After 8 months of wound care and parenteral nutrition, we decided to attempt primary wound closure because the patient required permanent oral restriction and total parenteral nutrition due to short bowel syndrome. A circular incision along the circumference of the exposed bowel allowed us to take a safe approach into the abdominal cavity. We removed the intestinal adhesions completely and resected the bowels, including the fistulas and anastomosed parts. Finally, the abdominal wall defect was reconstructed using the component separation technique, and the patient was discharged without an ostomy.

**Conclusions:**

Primary fascia closure for grade 4 open abdomen is hard, but leaving a long interval before radical surgery and applying pertinent wound management may help solve this adverse situation.

## Background

The open abdomen (OA) is a life-saving surgical procedure for abdominal rigidity due to severe abdominal trauma, sepsis, or abdominal compartment syndrome (ACS), which involves exposing the intra-abdominal organs to the external environment [1]. The management of patients with OA requires three fundamental steps: hemodynamic resuscitation, source control of abdominal sepsis, and delayed abdominal closure [2].

The severity of OA is classified according to the presence of enteral effluent contamination, the degree of adhesion, and enteroatmospheric fistula (EAF) formation [3]. The most severe, grade 4, is defined as “frozen OA with adherent/fixed bowel, unable to close surgically, with or without fistula”; thus, only delayed skin closure with a skin flap or a skin graft are possible closure options [2]. There are few reported grade 4 OA cases with successful primary fascia closure and the complete removal of enteral adhesions [4][5][6]. To the best of our knowledge, no case has been reported in the literature of primary fascia closure for grade 4 OA after repeated laparotomy.

We report a case of Björck grade 4 OA after repeated laparotomy for adhesive small bowel obstruction (SBO), which was managed with complete adhesiolysis. Appropriate wound management with negative pressure wound therapy (NPWT), a sufficient resting period to let the peritonitis subside, and nutritional support made it possible to perform a successful elective primary fascia closure.

## Case presentation

A 52-year-old man suffered acute abdominal pain while on a flight to the European continent. Upon landing, he was transferred to a regional hospital, where an emergency laparotomy was performed. He had undergone open laparotomy twice before for a complicated appendectomy and recurrent ileus, after which he was diagnosed with adhesive SBO. The first operation revealed several serious bowel adhesions, which were excised. Early recurrent ileus occurred a few days after the first surgery, and more than 10 repeated laparotomies were required. According to the hospital report where he was treated previously, the surgeon decided to construct a terminal jejunostomy due to ubiquitous, unsolvable, strong adhesions in the sixth operation. Finally, OA was selected as a solution. Considering his situation, the patient chose to return to his home country, so he was transferred to our institution.

**Fig. 1:**
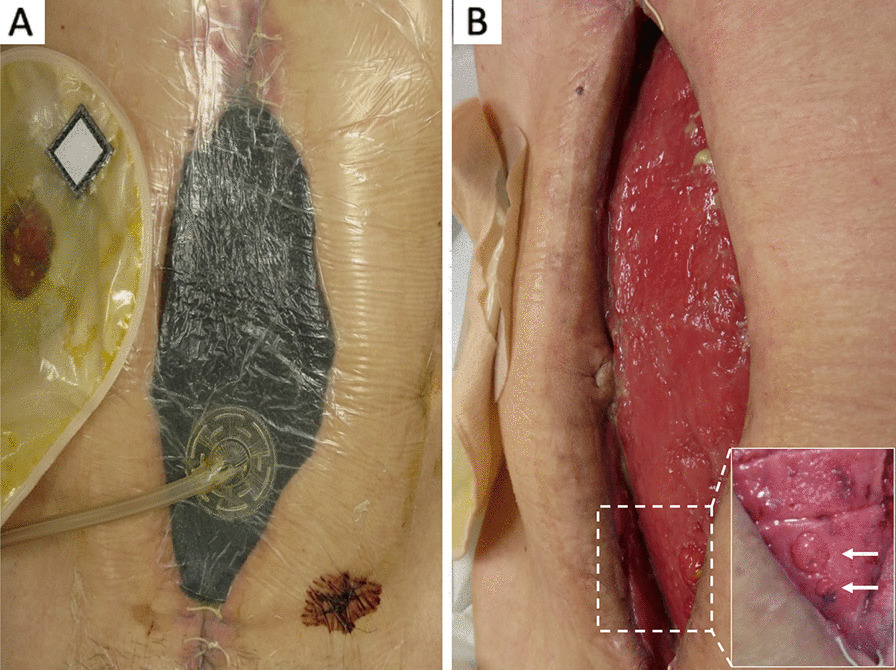
Abdominal findings on admission to our hospital. **A** Negative pressure wound therapy (NPWT) was introduced, and jejunostomy was performed. **B** The exposed bowels were covered by granulation tissue, with a crevice between the abdominal wall and bowels and two enteroatmospheric fistulas (EAFs) (arrows)

**Fig. 2:**
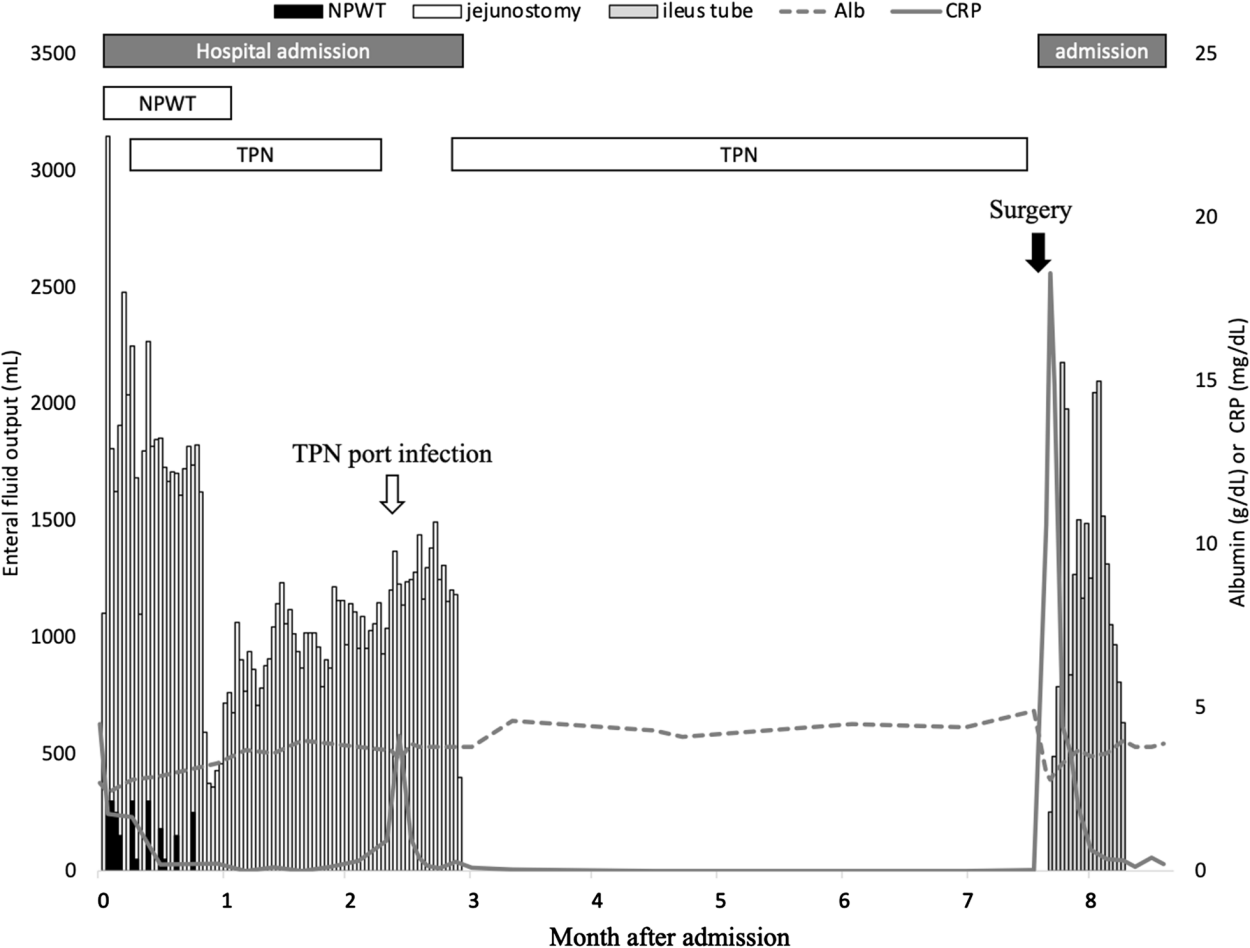
Daily fluid output and laboratory data after the initial admission. *NPWT* negative pressure wound therapy, *TPN* total parenteral nutrition, *Alb* serum albumin, *CRP* C-reactive protein

**Fig. 3:**
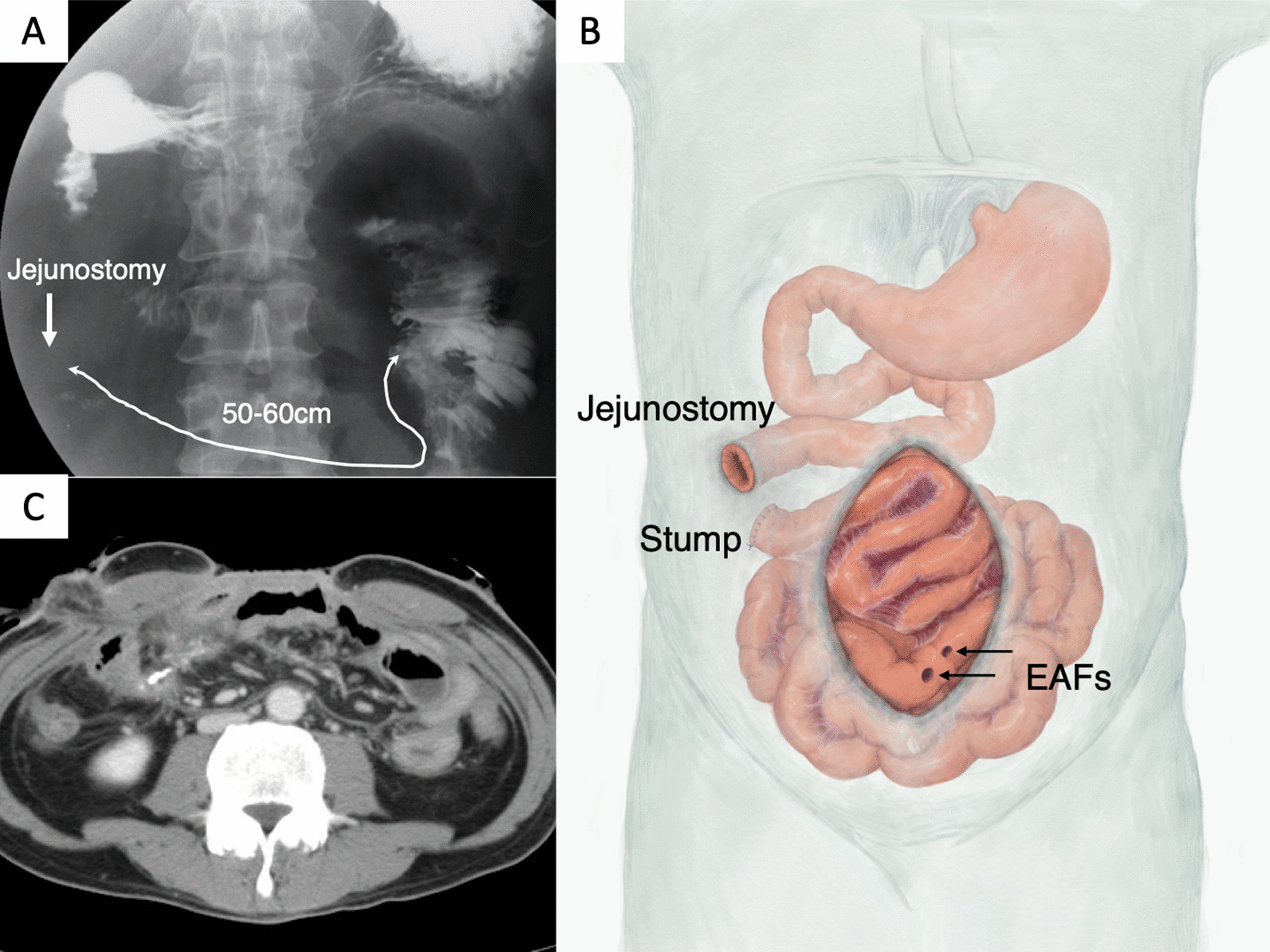
Examination findings on admission. **A** The barium swallow test revealed that only 50–60 cm of the jejunum length was preserved on the proximal side of the jejunostomy. **B** Diagram of abdominal findings on admission. **C** Edema of exposed bowels was found on CT

NPWT was introduced for OA on admission to our hospital, and terminal jejunostomy was performed in the right upper abdominal quadrant (Fig. 1A). Granulating tissue covered the adhered bowels almost completely, but it had not yet spread to the crevice between the organs and abdominal wall (Fig. 1B). Two EAFs were observed close to the lower edge of the exposed bowel (Fig. 1B, square); therefore, we diagnosed his condition as a Björck grade 4 open abdomen. The daily fluid output from EAF and terminal jejunostomy was 200–300 mL and 1000–3000 mL, respectively (Fig. 2). Laboratory examination revealed persisting abdominal inflammation (C-reactive protein level, 4.5 mg/dl), anemia (hemoglobin level, 10.5 g/dl), and hypoalbuminemia (serum albumin level, 2.7 g/dl). The gastrografin swallow test revealed that only 50 to 60 cm of the jejunum was left on the proximal side of the terminal jejunostomy (Fig. 3A, B), and abdominal enhanced computed tomography (CT) revealed a defect of the abdominal wall with intestinal edema and ascites, reflecting peritonitis (Fig. 3C).

**Fig. 4:**
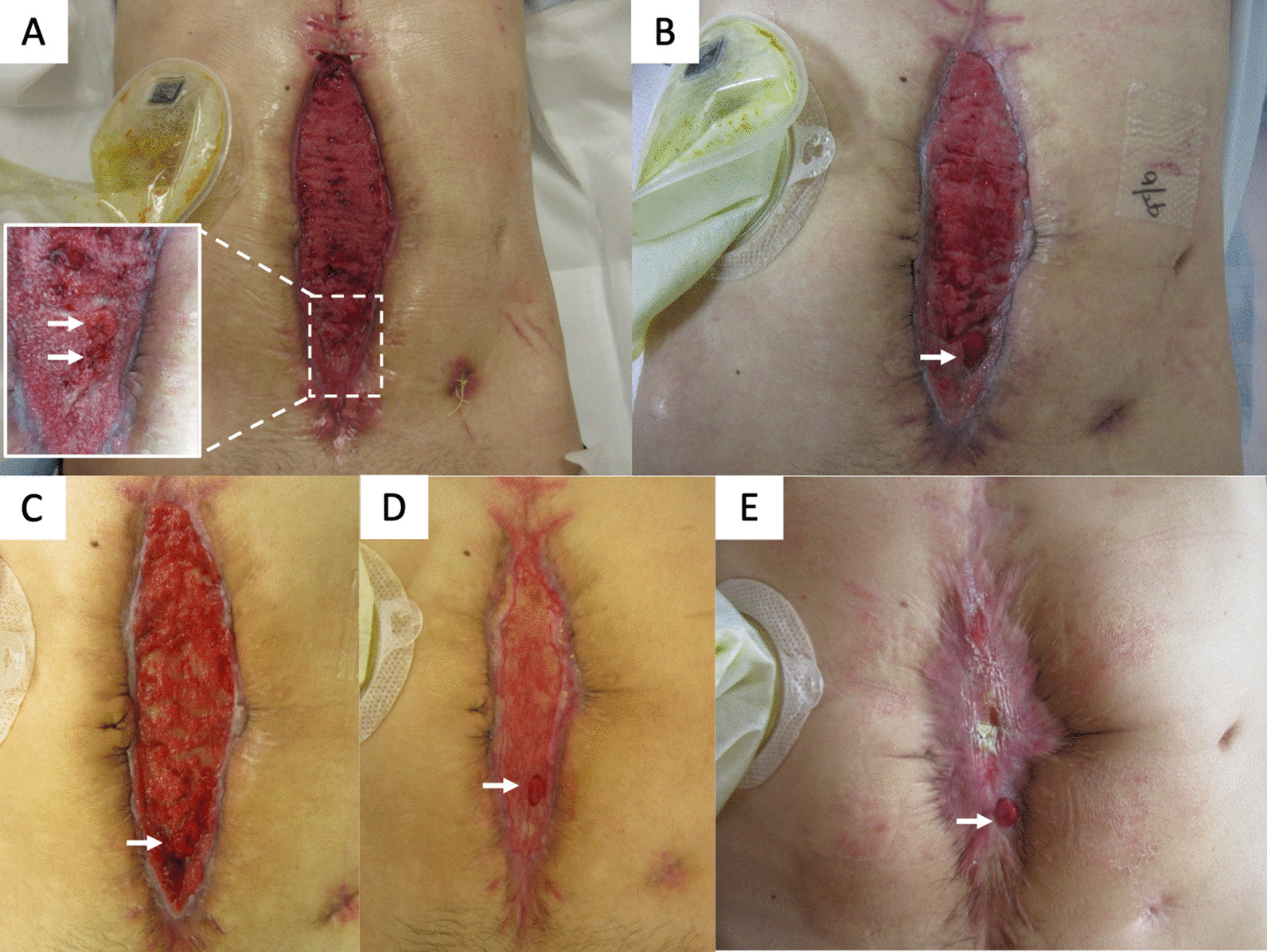
Time-dependent evolution of the abdominal wound findings. **A** 3 weeks after admission: granulation tissue covered the crevice between the abdominal wall and the intestines. **B** 4 weeks, **C** 8 weeks, and **D** 12 weeks after admission. **E** 8 months after admission. The open abdomen was completely covered by scar tissue, but the EAF persisted like an ostomy (arrow)

As a surgical intervention was impossible considering his clinical status, we decided to perform continuous NPWT management and total parenteral nutritional (TPN). After 3 weeks of NPWT, the granulating tissue had gradually protruded to cover the crevice between the intestine and the abdominal wall, but the EAFs did not close (Fig. 4A). The inflammation was resolved, but high enteral fluid loss from proximal jejunostomy and short bowel syndrome (SBS) required continuous intravenous fluid and nutrition replacement. Spontaneous EAF closure could not be expected; therefore, we shifted the treatment strategy to elective surgical intervention after a long resting interval of more than half a year for wound healing and nutritional recovery. Thanks to the Wound Ostomy Continence (WOC) nursing team’s intensive wound management, the OA surface shrank. The wound edge was covered by desiccated granulation tissue after a 2-month hospitalization (Fig. 4B, C). A small EAF was closed by NPWT within 3 weeks; however, the other EAF did not close, and a mucosal prolapse became evident as an ostomy (Fig. 4D). The patient was temporarily discharged from the hospital after a 3-month stay and went home with TPN via a central line and an oral elemental diet, allowing him to wait long enough until the surgical intervention. Eight months later, the wound granulation tissue had desiccated almost completely (Fig. 4E), and the patient’s nutritional status had recovered to a normal level with the only exception of a mild transaminase level elevation due to TPN.

**Fig. 5:**
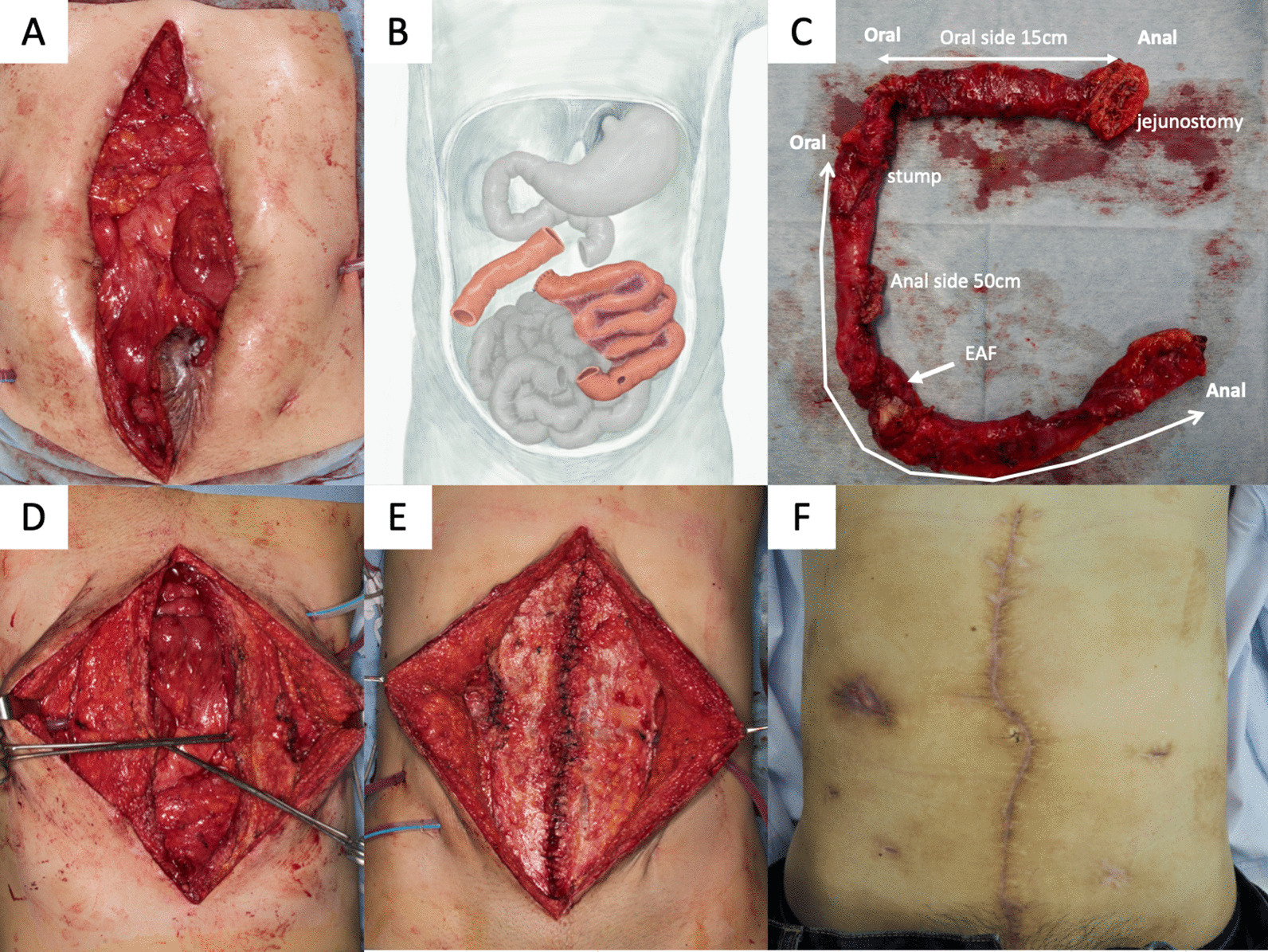
Operative findings. **A** Complete adhesiolysis was achieved using a circular incision. **B** Surgical diagram. Two segments of the small intestines were removed, and both ends were anastomosed. **C** Findings of resected bowels: 15 cm of the proximal jejunum and 50 cm of the distal jejunum, including the EAF, were resected. **D**, **E** The abdominal wall defect was reconstructed using the component separation technique. **F** Abdominal findings two months after radical surgery

We planned a two-team surgical approach, with a gastrointestinal surgeon and a plastic surgeon for radical surgery of the severely adhesive bowels and the large defect of the abdominal wall. A circular surgical incision along the abdominal wound circumference was made to avoid injury to the frozen adhesive intestine (Fig. 5A). After reaching the abdominal cavity, diffuse predictable intestinal adhesion was observed involving the whole small intestine. However, as we had provided enough time for the resolution of the peritoneal inflammation, we were able to achieve complete adhesiolysis, and we resected the frozen adherent bowel segment, including the EAF foramen (Fig. 5B, C). The proximal site terminal jejunostomy was also removed and anastomosed. The remaining length of the small intestine was approximately 215 cm, meaning the SBS was also cured. Finally, the large abdominal wound defect was reconstructed using a component separation technique (Fig. 5D, E). A long intraluminal enteral drainage tube was inserted into the small intestine during surgery. The postoperative course presented no serious evident morbidity. There was no evidence of small bowel dilatation on the radiographs, though a high drainage output from the drainage tube continued for approximately 2 weeks (Fig. 2). The long drainage tube removal had to wait 3 weeks before the patient could start a regular oral diet and return to a routine without TPN and ostomy (Fig. 5F). He did not present any recurrent ileus during the last 3 years of follow-up.

## Discussion and conclusion

This report demonstrates that primary wound closure with complete adhesiolysis can be successful in a patient with severe adhesive Björck grade 4 OA with EAF after over 10 reoperative surgeries for SBO. We emphasize that the following two key management points made it possible to solve this adverse situation: pertinent wound management, including NPWT, and a long interval prior to elective surgery (> 6 months).

Adhesive SBO is not usually considered an indication for OA management. The World Society for Emergency Surgery (WSES) guidelines hardly recommend OA application for severe peritonitis or extensive visceral edema due to a concern about possible ACS [1]. In our patient, repeated laparotomy was carried out after emergency laparotomy for adhesive SBO. Thus, the iatrogenic risk of ACS due to enteral edema was considered an indication for OA. Though the SBO was stabilized by OA and proximal-site jejunostomy, the patient suffered from a large disfigured abdominal wound and permanent oral restriction due to SBS.

Managing a patient with OA is a great challenge, and the best way to manage a case remains unclear. A classification system was proposed by Björck et al. to help clarify the challenges related to OA [3]. OA grade is determined by the presence or absence of a wound and bowel adherence, wound fixity (which means lateralization of the abdominal wall), and fistula formation.

The most severe, grade 4, OA is defined as adherent/fixed bowel unable to be closed surgically, with or without fistula, and the key feature of its management is prevention [3]. However, once this adverse situation emerges, the fascia and skin cannot usually be dissected from the visceral tissue, so primary fascia closure cannot be performed. Therefore, closure using skin grafts is performed after granulation tissue development [2]. A scoring system named the Open Abdomen Fascia Closure (OAFC) score was proposed by Yetisir et al. to help distinguish these patients from OA patients whose fascia should be closed [2] The OAFC score includes age, malignancy, and Sequential Organ Failure Assessment (SOFA) score. Our patient had a relatively young age and no malignant disease or organ failure, so his OAFC score was 0; therefore, fascial closure could be considered. The patient also suffered SBS since the remaining length of the functional small intestine was only 50 cm, and this fact also challenged us to consider a complicated radical surgery.

A few cases of primary fascia closure for Björck grade 4 OA have been reported previously. Yetisir et al. wrote up a case series on the operative management of Björck grade 4 OA [4]. In their reports, four of five cases had been treated by a skin graft or skin flap; however, one case in which radical surgery was performed was included. That patient developed OA after anastomotic leakage of left colectomy, and the surgery details (using laparoscopic lateral approach) were reported separately [6]. Eğin et al. reported another case of primary fascia closure for OA with a spontaneously developed EAF after Hartmann’s procedure for colostomy closure and incisional hernia. Both cases were graded 4 on the Björck scale. However, our subject seemed to present a more severe degree of bowel adhesion due to repeated laparotomy for adherent SBO and a larger abdominal wall wound defect.

The key surgical management points are delayed primary closure with an adequate interval to ensure the eradication of abdominal sepsis and a lateral approach, which means a circumferential incision around the granulated tissues [7][8]. We safely reached the abdominal cavity, avoiding injury of the intestine by using the lateral approach, and we were able to remove the severe enteral adhesion. Abdominal wall defect reconstruction was also an essential point of the surgery. Primary fascia closure of a large abdominal wall defect usually requires a muscle-cutaneous flap; therefore, a two-team approach with a plastic surgeon is recommended. In our case, we did not need a muscle-cutaneous flap due to the shrinkage of the OA wound, and we performed reconstruction using the component separation technique. Thus, our approach of delayed surgical management also improved the patient’s quality of life.

In conclusion, Björck grade 4 OA is a severe surgical complication, and prevention is essential for reducing the related morbidity. However, if a patient suffers such an adverse event, appropriate wound care and delayed surgery may contribute to the patient’s satisfactory recovery.

## Data Availability

Data sharing is not applicable to this article as no datasets were generated or analyzed during the current study.

## Abbreviations

ACS: Abdominal compartment syndrome
CT: Computed tomography
EAF: Enteroatmospheric fistula
NPWT: Negative pressure wound therapy
OA: Open abdomen
OAFC score: Open abdomen fascia closure score
SBO: Small bowel obstruction
SBS: Short bowel syndrome
SOFA score: Sequential organ failure assessment score
TPN: Total parenteral nutrition
WSES: World Society for Emergency Surgery
WOC: Wound ostomy continence
